# The characterization of dysphagia in COVID‐19 rehabilitation patients: An inpatient rehabilitation cohort study

**DOI:** 10.1002/pmrj.70113

**Published:** 2026-04-09

**Authors:** Benjamin K. Petrie, Christopher W. Lewis, Sean Dreyer, Prakash Jayabalan

**Affiliations:** ^1^ Shirley Ryan AbilityLab Chicago Illinois USA; ^2^ Department of Physical Medicine & Rehabilitation Northwestern Feinberg School of Medicine Chicago Illinois USA; ^3^ Department of Biomedical Informatics & Medical Education University of Washington Medicine Seattle Washington USA

## Abstract

**Background:**

Severe COVID‐19 infection requiring hospitalization is associated with oropharyngeal dysphagia, yet trajectories in the inpatient rehabilitation facility (IRF) setting remain undercharacterized.

**Objective:**

To investigate the prevalence, risk factors, recovery likelihood, and evolution of dietary requirements of patients with dysphagia due to severe COVID‐19 infection admitted to IRF.

**Design:**

Retrospective cohort study.

**Setting:**

Three free‐standing urban IRFs.

**Patients:**

Two hundred forty eight patients with acute symptomatic COVID‐19 admitted to IRF between April 2020 and February 2021. Dysphagia was defined by abnormalities on videofluoroscopic swallow study, Functional Oral Intake Scale, or Mann Assessment of Swallowing Ability. Patients with neurologic disease, nil per os status on IRF admission, or indeterminate dysphagia status were excluded.

**Interventions:**

Multidisciplinary IRF dysphagia treatment, per standard of care.

**Main outcome measures:**

Dysphagia prevalence and resolution, dietary consistencies, and Functional Independence Measure scores.

**Results:**

Of the included patients, 46.0% had dysphagia on admission (*n* = 114), most commonly oropharyngeal (89.7%), followed by oral (8.6%) and pharyngeal (1.7%). Dysphagia resolved during an average 3‐week IRF stay in 43.5% of cases. Higher Functional Independence Measure comprehension scores were associated with lower likelihood of dysphagia (*p* = .002) and higher likelihood of resolution (*p* = .021). Discharge to home was common in both those with persistent and resolved dysphagia (86.5% vs. 88.6%). Additionally, 92.1% (*n* = 105) of patients with dysphagia on admission were discharged on a normal or International Dysphagia Diet Standardisation Initiative Level 6/0 diet.

**Conclusions:**

In this large study of dysphagia in patients with COVID‐19 requiring IRF, a strong, novel association between cognitive function and both dysphagia incidence and recovery was observed in patients without discrete neurologic injury. Although persistent dysphagia was common at discharge, most patients transitioned to a normal or near‐normal diet.

## INTRODUCTION

SARS‐COV‐2 is the virus that causes COVID‐19). This disease that the World Health Organization classified as a global pandemic in March 2020 has been associated with a spectrum of neurological manifestations, including the development of dysphagia.^1^ Estimates of the prevalence of dysphagia after COVID‐19 infection in hospitalized patients have been variable, although larger studies approximate it to be 30%.^2^, ^3^

Multiple factors are related to the development of dysphagia in critically ill patients including prolonged hospitalization and intensive care unit (ICU) stay, intubation (particularly with repeat intubation), heart failure, and impaired functional status; many of these risk factors are present in those with COVID‐19 infection requiring hospitalization.^4^ In individuals with COVID‐19, direct viral neuroinvasion occurs and may play a role in dysphagia development as well.^1^ Regardless of the underlying mechanism, acute care studies have found dysphagia to be associated with increased risk of aspiration pneumonia, prolonged hospitalization, and increased mortality.^5^

During the COVID‐19 pandemic, physiatrists, nurses, and therapists were tasked with managing dysphagia in this new cohort of patients without robust literature guiding their decisions. There are a few small‐scale studies that investigate COVID‐19 infection‐associated dysphagia risk factors and recovery, but these studies are uniformly in the ICU or general hospital setting, not inpatient rehabilitation facilities (IRF).^3^, ^6^, ^7^, ^8^, ^9^, ^10^, ^11^ Given that dysphagia is associated with increased health care costs, worse quality of life, more frequent transfers from IRF, and decreased likelihood of discharging home from an IRF, identifying individuals at risk for dysphagia development and expected recovery is important for resource planning.^12^, ^13^

More broadly, health care‐associated infections, including COVID, present a significant challenge in rehabilitation settings, often requiring isolation precautions that can disrupt standard therapy delivery. However, rehabilitation programs can be effectively adapted to patients with infectious conditions, allowing for functional progress with proper isolation precautions.

The present retrospective study investigated COVID‐19 infection‐associated dysphagia in hospitalized patients requiring inpatient rehabilitation. Our objectives were to identify risk factors for dysphagia development and resolution and to compare dysphagia diets on IRF admission and discharge, focusing on percentage with dysphagia resolution during their IRF stay. We hypothesized that most individuals with dysphagia related to COVID‐19 would make a full recovery of the swallowing disorder in the IRF and certain domains of the Functional Independence Measure (FIM) would be associated with recovery.

## METHODS

### Setting

This study included a retrospective review of patient records at 3 free‐standing IRFs located in a large urban city. As required for admission to an IRF, patients were scheduled to participate in a minimum of 900 minutes of therapy per week.^14^ This study was approved by the local institutional review board. Due to the nature of the study, which involved secondary use of deidentified patient data, the requirement for individual informed consent was waived by the institutional review board.

### Study design

This was a retrospective cohort study of patients admitted to an IRF following initial acute hospitalization for COVID‐19 infection between April 14, 2020 and February 21, 2021. COVID‐19 was the primary reason for admission in all patients, with Centers for Medicare and Medicaid Services Impairment Groups Pulmonary, Debility, and Medically Complex Conditions. Chart review of electronic health records of both IRF and acute care was performed to collect demographic information, medical history, acute care course, complications during acute care or inpatient rehabilitation, and rehabilitation outcomes. Patients were excluded if they had a preexisting neurological condition (eg, acute stroke) that could cause dysphagia, developed a neurological condition that could cause dysphagia during acute care or IRF admission (independent of COVID‐19 infection), were nil per os (NPO) on admission to IRF, or had an indeterminate dysphagia status. Patients who were NPO on admission to IRF were excluded to avoid erroneously including patients made NPO for nondysphagia‐related reasons, such as intestinal ischemia, esophageal perforation, gastric/esophageal damage, or gastrointestinal bleeding. Figure 1 outlines the study cohort selection process.

**FIGURE 1:**
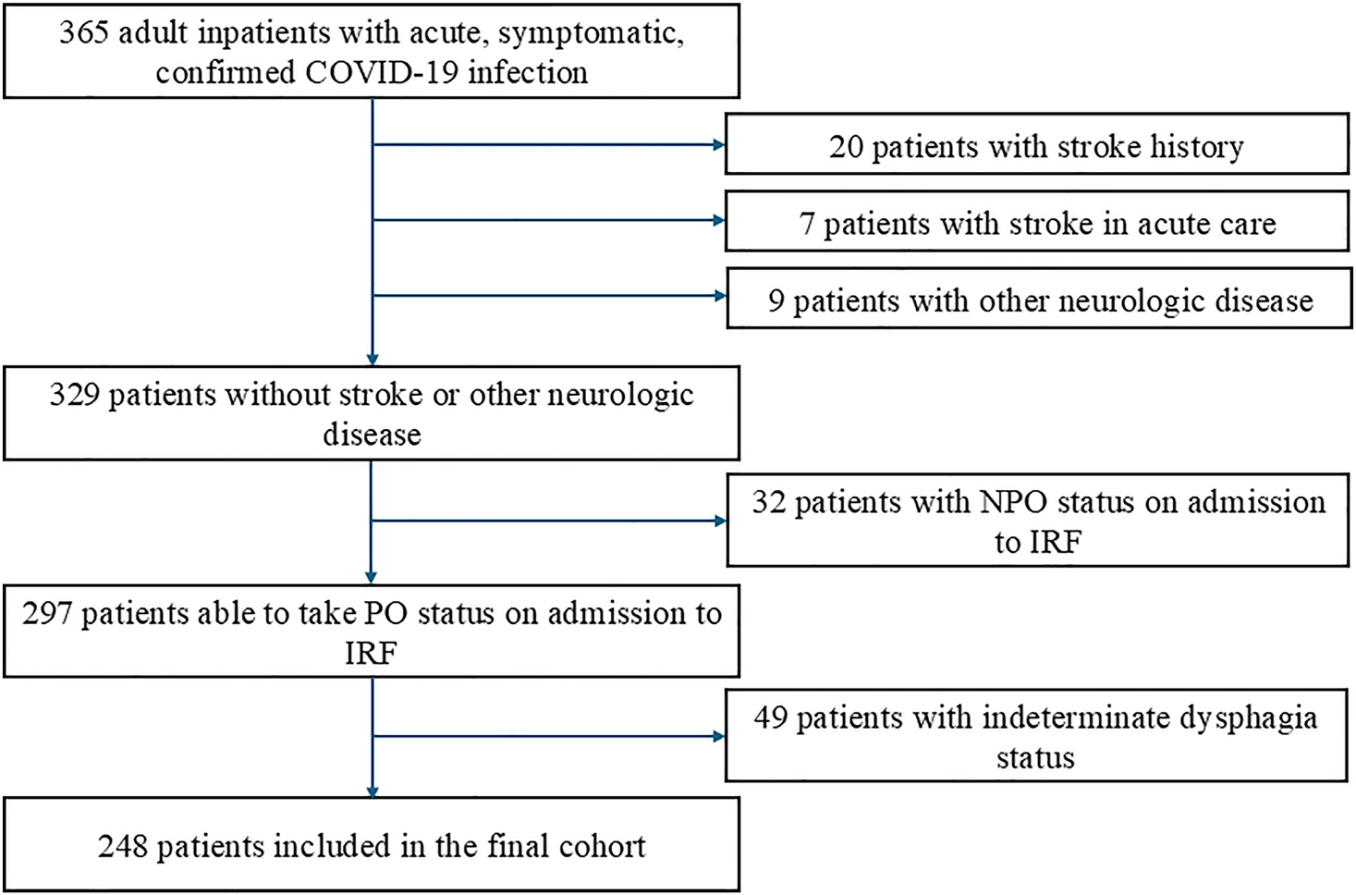
Flow chart of patient selection process. Patients with other neurologic disease included patients with multiple sclerosis, Parkinson disease, and central nervous system malignancy. IRF, inpatient rehabilitation facility; NPO, nil per os; PO, per os.

### Dysphagia assessment

All patients with COVID‐19 infection were screened for dysphagia on admission to the IRF, and they were subsequently treated for this if present. Standardized dysphagia assessment tools were used for assessment and dysphagia monitoring. These tools included the Mann Assessment of Swallowing Ability (MASA), Functional Oral Intake Scale (FOIS), and videofluoroscopic swallow study (VFSS). The MASA is a 24‐item scale that uses a 5‐to‐10‐point scoring system, with a total score of 200 points and a cutoff for mild dysphagia of <177.^15^ The FOIS is a 1‐to‐7‐point scoring system with a score of 1 being NPO and a score of 7 being a normal solid/liquid diet.^16^ VFSS results were broken down into normal swallowing, oral dysphagia, pharyngeal dysphagia, and oropharyngeal dysphagia.

VFSS is the gold standard for dysphagia diagnosis. Patients were considered to have dysphagia if they had evidence of dysphagia on VFSS on IRF admission.^17^ Given that VFSS can be difficult to do precipitously in IRF, if there was no VFSS result, a MASA of <177 or a FOIS of <7 on admission to IRF was considered consistent with dysphagia. An IRF VFSS with no evidence of dysphagia, or MASA >177 and FOIS 7 in those without VFSS, was considered consistent with normal swallowing ability.

VFSS performed in acute care was used to identify dysphagia type, but patients with dysphagia by VFSS in acute care with normal swallowing on IRF admission (ie, normal VFSS and/or normal MASA/FOIS) were not considered to have dysphagia. Given that there are reasons other than dysphagia a patient may be placed on a modified International Dysphagia Diet Standardisation Initiative (IDDSI) diet, the presence of being admitted to IRF on a modified IDDSI diet in and of itself was not a criterion for dysphagia classification (Figure S1).

Patients were classified as having indeterminate dysphagia status if VFSS was not performed on admission to IRF and they had missing MASA and FOIS scores. This may have been due to cognitive limitations, medical instability, short length of stay, or other reasons. As discussed previously, these patients were excluded from the study.

Dysphagia resolution was concluded if there was evidence of resolution of dysphagia on repeat VFSS. If repeat VFSS was not performed, speech‐language pathologists determined when a patient's dysphagia had resolved. The measures that were evaluated by speech‐language pathologists included MASA (>177), FOIS (=7), or tolerance of a IDDSI solid Level 7 and liquid Level 0 diet.

### Functional and cognitive assessment

Functional status was assessed using FIMs, which are a standardized set of 18 items scored on a 7‐point scale, covering motor and cognitive domains. FIM scores were extracted from IRF admission and discharge assessments. In addition to the cognitive FIM scores, patients were also tracked with Orientation Log, a standardized 10‐item rating scale out of 30 used to assess cognitive function over time.^18^

### Statistical analysis

Initially, descriptive patient characteristics were calculated for the full sample using mean (±SD) for continuous variables and counts (percentage) for categorical variables. Dysphagia prevalence in the full sample was calculated. The sample was divided into groups with and without dysphagia and patient characteristics were compared between the two groups. *T*‐tests were used to compare continuous variables, *χ* ^2^ test was used to compare categorical variables, and Mann–Whitney *U* tests were used to compare FIMs and Orientation Logs between these two groups. Patients with dysphagia were then subgrouped into those with persistent dysphagia and dysphagia resolution and the aforementioned analyses were used to compare these two subgroups. Patient MASA scores, FOIS scores, VFSS results, and IDDSI variables were descriptively compared on admission to discharge. Logistic regression was used with explanatory variables found to be significant in crude analyses using dysphagia development or resolution as response variables, controlling for patient age on admission and gender. A *p* < .05 was considered statistically significant. R version 4.2.2 was used for the statistical analysis.

## RESULTS

### Patient sample

Patients with COVID‐19 infection who were subsequently admitted to IRF were considered by inclusion criteria (*n* = 365) (Figure 1). Of those, 20 were excluded due to a history of stroke, and 7 were excluded due to experiencing a stroke in acute care. Subsequently three patients were excluded due to a history of Parkinson disease, four were excluded due to history of multiple sclerosis, and two were excluded due to history of central nervous system cancer. In addition, 32 patients were excluded due to being NPO on admission to IRF, and 49 patients were excluded due to indeterminate dysphagia status on admission to IRF, which led to an analysis cohort of 248 patients. Patients primarily came from Centers for Medicare and Medicaid Services Impairment Groups Pulmonary, Debility, and Medically Complex Conditions.

### Patient demographics and dysphagia prevalence

The mean age of the analysis cohort was 63.0 ± 13.5 years, ranging from 23 to 92 years; 66.3% of patients were male and 33.7% were female. The mean acute care length of stay was 26.6 ± 15.9 days, ICU length of stay was 13.6 ± 11.9 days, and 74.6% of patients were intubated at least once. Patients stayed on average 19.3 ± 9.9 days in IRF. On admission to IRF, 114 (46.0%) of included patients had evidence of dysphagia.

### Characteristics of patients with and without dysphagia at IRF admission

Referring to Table 1, compared to those without dysphagia (*n* = 134), patients with dysphagia (*n* = 114) had longer stays in acute care (32.6 ± 18.4 days vs. 22.7 ± 12.8 days, *p* = .006) and the ICU (18.4 ± 14.9 days vs. 10.7 ± 8.7 days, *p* = .024). Admission FIM Comprehension scores (4.9 ± 1.6 vs. 5.7 ± 1.3, *p* = .002) and Orientation Log scores (27.4 ± 3.5 vs. 24.9 ± 7.0, *p* = .005) were both lower in patients with dysphagia compared to those without. Additionally, upper body dressing (3.2 ± 2.3 vs. 3.7 ± 1.73, *p*  <.001), lower body dressing (2.5 ± 2.2 vs. 2.9 ± 1.6, *p*  <.001), and chair transfers (2.5 ± 1.0 vs. 3.0 ± 0.7, *p* = .001) admission FIMs were also lower in those with dysphagia compared to those without dysphagia.

**TABLE 1:** Patient demographic and clinical characteristics in patients without and with dysphagia.

Variable		No dysphagia (*n* = 134)	Dysphagia (*n* = 114)	*p* value
Age, mean ± standard deviation		63.5 ± 13.5	62.5 ± 13.5	.557
Body mass index		33.6 ± 9.1	30.5 ± 7.3	.061
Male, %		62.9%	70.2%	.284
Race	Caucasian	46.3%	38.6%	.185
	Black	14.9%	20.2%	.185
	American Indian	0%	1.8%	.185
	Asian	10.0%	8.8%	.185
	Native Hawaiian	0%	0%	.185
	Hispanic	21.0%	27.2%	.185
	Other	8.2%	3.5%	.185
Acute care length of stay, mean ± standard deviation		22.7 ± 12.8	32.6 ± 18.4	.006*
ICU length of stay		10.7 ± 8.7	18.4 ± 14.87	.024*
Intubated once or more in acute care		68.6%	84.1%	.103
IRF admission O_2_ rate		1.2 ± 1.4	1.1 ± 1.3	.537
IRF admission comprehension FIM		5.7 ± 1.3	4.9 ± 1.6	.002*
IRF admission orientation Log		27.4 ± 3.5	24.9 ± 7.0	.005*
IRF admission upper body dressing FIM		3.7 ± 1.7	3.2 ± 2.3	<.001*
IRF admission lower body dressing FIM		2.9 ± 1.6	2.5 ± 2.2	<.001*
IRF admission chair transfer FIM		3.0 ± 0.7	2.5 ± 1.0	.001*
IRF admission walk 10 feet FIM		3.0 ± 1.0	2.8 ± 0.8	.081
IRF admission walk 50 feet FIM		3.3 ± 0.9	3.0 ± 1.0	.223

*Note*: Comparison of patient demographic and clinical characteristics between those without and with dysphagia as above. A *p* < .05 was considered significant and demarcated with *.

Abbreviations: IRF, inpatient rehabilitation facility; FIM functional independence measure.

### Characteristics of patients with and without dysphagia resolution at IRF discharge

Seven patients were removed from this analysis given conflicting or incomplete discharge data (Tables 2 and 3). In those with dysphagia, 43.5% (*n* = 37) of individuals experienced resolution of dysphagia during IRF stay and 66.5% (*n* = 70) did not. Patients with dysphagia resolution were younger (59.7 ± 14.3 vs. 66.4 ± 10.7, *p* = .008) and had higher body mass index (33.0 ± 7.6 vs. 27.0 ± 6.0, *p* = .015) compared to those who did not. Compared to those with persistent dysphagia, the FIM Comprehension scores (6.2 ± 0.9 vs. 5.4 ± 1.2, *p* = .004) and Orientation Log scores (28.0 ± 1.9 vs. 24.1 ± 6.5, *p* = .027) at discharge were higher for those with resolved dysphagia. Lower body dressing (5.0 ± 1.2 vs. 4.3 ± 1.2, *p* = .005), upper body dressing (5.5 ± 0.8 vs. 5.0 ± 0.9, *p* = .002), and chair transfer (5.1 ± 1.1 vs. 4.5 ± 1.2, *p* = .005) FIMs were higher in those with dysphagia resolution compared to those without resolution. Both those with and without dysphagia resolution discharged home at high rates after IRF (88.6% vs. 86.5%, *p* = .998).

**TABLE 2:** Patient demographic and clinical characteristics in dysphagic patients with and without dysphagia resolution.

Variable		Dysphagia resolved (*n* = 37)	Dysphagia maintained (*n* = 70)	*p*‐value
Age, mean ± standard deviation		59.7 ± 14.3	66.4 ± 10.7	.008*
Body mass index		33.0 ± 7.6	27.0 ± 6.0	.015*
Male, %		71.4%	70.3%	1.000
Race	Caucasian	40.0%	37.8%	.286
	Black	25.7%	13.5%	.286
	American Indian	0%	5.4%	.286
	Asian	8.6%	10.8%	.286
	Native Hawaiian	0%	0%	.286
	Hispanic	22.9%	27.0%	.286
	Other	2.9%	5.4%	.286
Acute care length of stay, mean ± standard deviation		33.2 ± 19.2	32.1 ± 19.7	.867
ICU length of stay		17.7 ± 16.1	20.0 ± 14.9	.752
Intubated once or more in acute care, %		84.6%	78.6%	.965
IRF discharge O_2_ rate, mean ± standard deviation		0.5 ± 0.9	0.6 ± 1.3	.710
IRF discharge comprehension FIM		6.2 ± 0.9	5.4 ± 1.2	.004*
IRF discharge orientation Log		28.0 ± 1.9	24.1 ± 6.5	.027*
IRF discharge upper body dressing FIM		5.5 ± 0.8	5.0 ± 1.0	.005*
IRF discharge lower body dressing FIM		5.0 ± 1.2	4.3 ± 1.2	.002*
IRF discharge chair transfer FIM		5.1 ± 1.1	4.5 ± 1.2	.005*
IRF discharge walk 10 feet FIM		4.8 ± 1.2	4.3 ± 1.4	.075
IRF discharge walk 50 feet FIM		4.8 ± 1.1	4.5 ± 1.1	.298
Home discharge		88.6%	86.5%	.998

*Note*: Comparison of patient characteristics between patients with dysphagia with and without dysphagia resolution as above. A *p* < .05 was considered significant and demarcated with *.

Abbreviations: IRF, inpatient rehabilitation facility; FIM functional independence measure.

**TABLE 3:** Dysphagia type, dysphagia scores and diets.

Variable		Admission	Discharge
Dysphagia type, %	Oral	8.6	
	Pharyngeal	1.7	
	Oropharyngeal	89.7	
Liquids	0	70.18	95.58
	1	0	0
	2	21.05	3.54
	3	7.02	0.88
	4	1.75	0
Solids	4	17.54	2.68
	5	8.77	3.57
	6	36.84	14.29
	7	36.84	79.46
FOIS	1	0	0
	2	0.93	0
	3	0.93	1.27
	4	1.85	0
	5	56.48	17.72
	6	25	17.72
	7	14.81	63.29
MASA, mean ± standard deviation		174.67 ± 12.08	186.14 ± 9.18

*Note*: Table summarizing dysphagia types, dysphagia scores, and diets at IRF admission and discharge. The diets represented here are consistent with the international dysphagia diet standardization initiative (IDDSI) classification.

Abbreviations: FOIS, functional oral intake scale; MASA, mann assessment of swallowing ability.

### Dysphagia type and diet characteristics

Referring to Figure 2, the majority of patients with dysphagia had oropharyngeal dysphagia (89.7%), and smaller proportions had oral (8.6%) and pharyngeal (1.7%) dysphagia. Additionally, 19.3% of patients with dysphagia had a gastronomy tube placed during their acute care hospitalization. On admission, the average MASA in patients with dysphagia was 174.7 ± 12.1, whereas at discharge the average MASA was 186.1 ± 9.2. At discharge, most patients with dysphagia were prescribed an IDDSI Level 7 solid diet (79.5%), followed by Level 6 solids (14.3%). Nearly all patients were prescribed an IDDSI Level 0 thin liquid diet (95.6%). Of those with dysphagia on admission, 92.1% were discharged on either an IDDSI Level 6 or 7 solid diet with an IDDSI Level 0 liquid diet.

**FIGURE 2:**
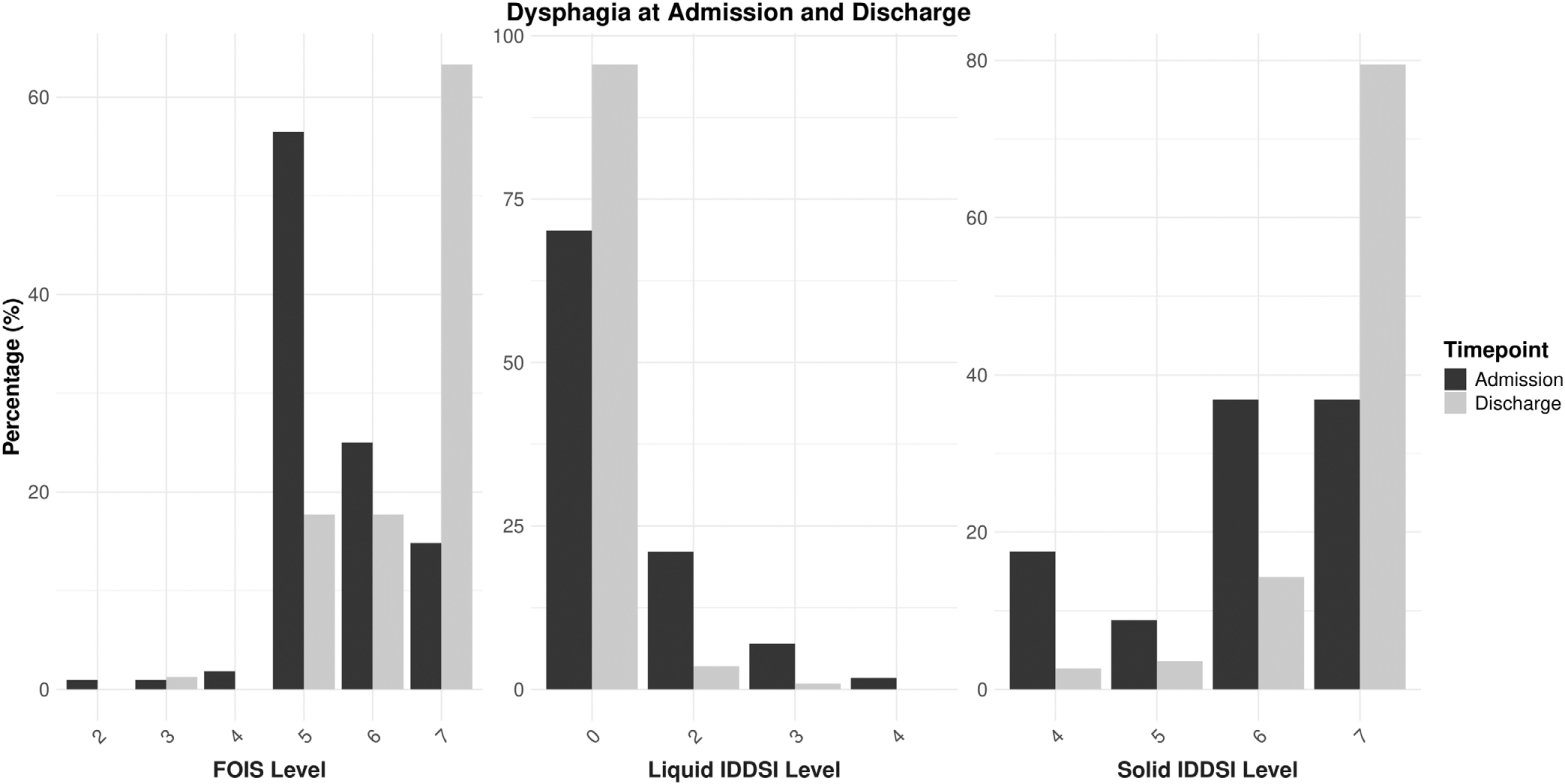
FOIS, Liquid IDDSI Level, and Solid IDDSI Levels distributions were compared on admission and discharge in patients with dysphagia who had complete admission and discharge data. FOIS, Functional Oral Intake Scale; IDDSI International Dysphagia Diet Standardization Initiative.

### Dysphagia development and resolution‐controlled analyses

Logistic regression was performed controlling for age and gender and considering the factors that were significant in the crude analyses described previously. Logistic regression demonstrated that the presence of dysphagia at admission was associated with a longer ICU length of stay (*p* = .011) and lower admission FIM Comprehension scores (*p* = .002). Logistic regression, also found that dysphagia resolution was associated with higher FIM Comprehension scores at discharge (*p* = .021).

## DISCUSSION

In this retrospective cohort study of 248 patients admitted to an IRF following COVID‐19 infection, the prevalence of dysphagia (usually mild, primarily oropharyngeal) was ∼50%, with a little over 40% of these individuals experiencing resolution of dysphagia at IRF discharge. Acute care length of stay for this population was ∼1 month, 2 weeks of which was spent in the ICU, and most had required intubation. Patients with dysphagia had significantly longer stays in the ICU and lower IRF admission FIM comprehension scores. Resolution of dysphagia was associated with higher discharge FIM comprehension scores. Almost 90% of the patients improved enough functionally to discharge to home following intensive rehabilitation, and >90% of those with dysphagia discharged on a normal or slightly modified diet (IDDSI Level 6/0).

To our knowledge, this 248‐patient study is the largest IRF study of dysphagia in patients with COVID‐19 infection. Compared to prior studies which only follow patients through their ICU or acute care hospitalization, this study adds a more complete characterization of COVID‐19 infection‐associated dysphagia in those that require IRF level of rehabilitation care.

The prevalence of patients developing dysphagia associated with severe COVID‐19 infection has previously been estimated at ∼30%.^2^, ^3^ There are several possible explanations for our prevalence being higher than other estimates. One possible explanation is that patients requiring IRF level of rehabilitation care are generally more functionally limited with longer acute care hospital stays, which has been previously associated with higher rates of dysphagia.^4^ Another explanation is that our universal screening for dysphagia identified patients who may have been missed in acute care. The higher prevalence of dysphagia than previously cited further emphasizes the importance of identifying these individuals early in their rehabilitation course.

In addition to the higher‐than‐expected prevalence of dysphagia in this population, the percentage of patients with residual dysphagia after IRF level of rehabilitation care was also higher than we expected. In the absence of a stroke or other discrete neurologic injury, we expected these individuals would fully recover with IRF rehabilitation. However, despite the high persistent dysphagia rate, 92.1% of patients with dysphagia on admission were discharged on at least an IDDSI Level 6/0 diet. A Level 6/0 diet can be considered a relatively mild form of dysphagia that can be easily managed with slight dietary modifications at home. Given that nearly 90% of patients discharged to home following IRF stay irrespective of dysphagia status, dysphagia persistence did not seem to be a barrier to discharge.

An interesting finding in this study was the establishment of a relationship between cognition and swallowing function in this population. The correlation between dysphagia and cognitive impairments has been described in other populations, although we believe this is a novel finding in those with COVID‐19 infection.^19^, ^20^ Thus, the presence of cognitive impairments in a patient with COVID‐19 infection may warrant a workup for occult dysphagia. The higher likelihood of dysphagia resolution in those with higher cognitive function further strengthens the relationship between these two impairments.

In crude analyses, dysphagia was more strongly associated with impairments in dressing and transfers than with ambulation, likely reflecting the higher cognitive demands of these activities of daily living tasks.^21^ This association disappeared in adjusted models, supporting the interpretation that cognitive impairment may mediate this relationship.

It has been shown consistently that longer lengths of stays in the ICU and general hospitalization are associated with dysphagia in this population.^4^ Although multiple prior studies demonstrate an association between intubation and dysphagia in this population, this was not observed in our study.^7^ One possible explanation for this is that patients were primarily assessed for dysphagia on admission to the IRF, whereas in other studies patients were actively screened for dysphagia in the ICU or general hospitalization.^9^, ^22^, ^23^ Research performed outside of the COVID‐19 infection population indicates that dysphagia secondary to intubation frequently does self‐resolve, in some patients within 24 hours of intubation.^24^, ^25^ It is therefore possible some patients had dysphagia resolution prior to IRF admission.

## LIMITATIONS

There are several important limitations of our study. The first is that this is an observational study, and we are unable to establish causation in any observed correlation. Unfortunately there were a sizable number of missing data entries in several variables specific to acute care, which may have influenced the patterns observed here. VFSS, the gold standard for dysphagia diagnosis, was not always performed on admission to IRF, leading to a possible misclassification bias in those where clinical rating scales were relied on. Similarly, VFSS was not always performed for all patients at discharge. Fiberoptic endoscopic evaluation of swallowing is another equivalent method of diagnosing dysphagia, although it was not used in this study because it is an aerosol generating procedure. It is possible the dysphagia results would be slightly different if fiberoptic endoscopic evaluation of swallowing were used instead of, or in addition to, VFSS. NPO patients were excluded to avoid including patients who were NPO for other reasons. However, this likely resulted in patients with severe dysphagia being excluded, potentially influencing the results. We studied only patients who were admitted to IRF following acute hospitalization, and these results, specifically resolution rates, should not be generalized to those that did not require, or otherwise did not receive IRF level of care following acute hospitalization discharge.

## CONCLUSION

We present, to our knowledge, the largest study characterizing dysphagia in COVID‐19 infected patients requiring IRF. Dysphagia was highly prevalent, and although persistent dysphagia was common, it was frequently mild and did not prevent discharge home. The association between cognition and dysphagia emphasizes the importance of appropriate screening for both dysphagia as well as cognitive deficits in this patient population.

## FUNDING INFORMATION

This study was funded by a Catalyst Grant from the Shirley Ryan AbilityLab. Dr Jayabalan receives support from the National Institute of Aging, Claude Pepper Center 1P30AG059988‐01A1.

## DISCLOSURE

The authors declare that there are no conflicts of interest regarding the publication of this manuscript. They have no relevant financial or nonfinancial interests to disclose.

This journal‐based CME activity is designated for 1.0 *AMA PRA Category 1 Credit* ^TM^. Effective January 2024, learners are no longer required to correctly answer a multiple‐choice question to receive CME credit. Completion of an evaluation is required, which can be accessed using this link, https://onlinelearning.aapmr.org/. This activity is FREE to AAPM&R members and available to nonmembers for a nominal fee. CME is available for 3 years after publication date. For assistance with claiming CME for this activity, please contact (847) 737–6000. All financial disclosures and CME information related to this article can be found on the Online Learning Portal (https://onlinelearning.aapmr.org/) prior to accessing the activity.

## Supporting information


Figure S1.

**Table S1.** Summary table of the key conclusions from the manuscript.

## Data Availability

The data that support the findings of this study are available from the corresponding author upon reasonable request.

## References

[1] Jha NK , Ojha S , Jha SK , et al. Evidence of coronavirus (CoV) pathogenesis and emerging pathogen SARS‐CoV‐2 in the nervous system: a review on neurological impairments and manifestations. J Mol Neurosci. 2021;71(11):2192‐2209. doi:10.1007/s12031-020-01767-6 PMC7814864 33464535

[2] Banari A , Aghaz A , Shahriyari A , Fakhimi F , Khoshgoftar M . The prevalence of dysphagia in patients with COVID‐19: a systematic review and meta‐analysis. Int J Health Med Res. 2023;2(7):172‐181.

[3] Asayama R , Tanaka‐Nishikubo K , Okada M , et al. Dysphagia in patients with severe COVID‐19: a retrospective study. Sci Rep. 2024;14(1):1‐5. 10.1038/s41598-024-57508-x PMC10957916 38514734

[4] Skoretz SA , Flowers HL , Martino R . The incidence of dysphagia following endotracheal intubation: a systematic review. Chest. 2010;137(3):665‐673. 10.1378/chest.09-1823 20202948

[5] Altman KW , Yu GP , Schaefer SD . Consequence of dysphagia in the hospitalized patient: impact on prognosis and hospital resources. Arch Otolaryngol Head Neck Surg. 2010;136(8):784‐789. 10.1001/archoto.2010.129 20713754

[6] Clayton NA , Freeman‐Sanderson A , Walker E . Dysphagia prevalence and outcomes associated with the evolution of COVID‐19 and its variants in critically ill patients. Dysphagia. 2024;39(1):109‐118. 10.1007/s00455-023-10598-7 PMC10781836 37347254

[7] Holdiman A , Rogus‐Pulia N , Pulia MS , Stalter L , Thibeault SL . Risk factors for dysphagia in patients hospitalized with COVID‐19. Dysphagia. 2023;38(3):933‐942. 10.1007/s00455-022-10518-1 PMC9483550 36109398

[8] Zayed AM , Afsah O , Elhadidy T , Abou‐Elsaad T . Screening for oropharyngeal dysphagia in hospitalized COVID‐19 patients: a prospective study. Eur Arch Otorrinolaringol. 2023;280(5):2623‐2631. 10.1007/s00405-022-07810-z PMC9821362 36607433

[9] Clayton NA , Walker E , Freeman–Sanderson A . Clinical profile and recovery pattern of dysphagia in the COVID‐19 patient: a prospective observational cohort within NSW. Aust Crit Care. 2022;36:262‐268. doi:10.1016/j.aucc.2022.01.001 PMC8758281 35177342

[10] Marchese MR , Ausili Cefaro C , Mari G , et al. Oropharyngeal dysphagia after hospitalization for COVID‐19 disease: our screening results. Dysphagia. 2022;37(2):447‐453. doi:10.1007/s00455-021-10325-0 PMC8222948 34165644

[11] Archer SK , Iezzi CM , Gilpin L . Swallowing and voice outcomes in patients hospitalized with COVID‐19: an observational cohort study. Arch Phys Med Rehabil. 2021;102(6):1084‐1090. doi:10.1016/j.apmr.2021.01.063 PMC7846878 33529610

[12] Castagna A , Ferrara L , Asnaghi E , Colombo V , Rega V , Fiorini G . Post‐stroke dysphagia rehabilitation in the old and oldest old: outcome and relevance for discharge home. Int J Rehabil Res. 2020;43(1):55‐61. 10.1097/MRR.0000000000000385 31714298

[13] A Practical Approach to Interdisciplinary Complex Rehabilitation E‐Book: A Google Books. Accessed April 26, 2024. https://books.google.com/books?hl=en&lr=&id=QDBcEAAAQBAJ&oi=fnd&pg=PA185&dq=dysphagia+management+benefits+from+an+inpatient+interdisciplinary+rehabilitation+team&ots=_wQDAClX5G&sig=DzIG6Sz3Orj31iY41xkrvceKXaA#v=onepage&q=dysphagia%20management%20benefits%20from%20an%20inpatient%20interdisciplinary%20rehabilitation%20team&f=false

[14] Forrest G , Reppel A , Kodsi M , Smith J , Enix D . Inpatient rehabilitation facilities: the 3‐hour rule. Medicine. 2019;98(37):e17096. 10.1097/MD.0000000000017096 PMC6750298 31517835

[15] Ohira M , Ishida R , Maki Y , et al. Evaluation of a dysphagia screening system based on the Mann assessment of swallowing ability for use in dependent older adults. Geriatr Gerontol Int. 2017;17(4):561‐567. 10.1111/ggi.12755 27195778

[16] Crary MA , Carnaby Mann GD , Groher ME . Initial psychometric assessment of a functional oral intake scale for dysphagia in stroke patients. Arch Phys Med Rehabil. 2005;86:1516‐1520. 10.1016/j.apmr.2004.11.049 16084801

[17] Kim J , Oh BM , Kim JY , Lee GJ , Lee SA , Han TR . Validation of the videofluoroscopic dysphagia scale in various etiologies. Dysphagia. 2014;29(4):438‐443. doi:10.1007/s00455-014-9524-y 24658847

[18] Penna S , Novack TA . Further validation of the orientation and cognitive logs: their relationship to the mini‐mental state examination. Arch Phys Med Rehabil. 2007;88(10):1360‐1361. 10.1016/j.apmr.2007.07.005 17908584

[19] Castagna A , Ferrara L , Asnaghi E , Rega V , Fiorini G . Functional limitations and cognitive impairment predict the outcome of dysphagia in older patients after an acute neurologic event. NeuroRehabilitation. 2019;44(3):413‐418. 10.3233/NRE-182635 31177241

[20] Maniaci A , Lechien JR , La Mantia I , et al. Cognitive impairment and mild to moderate dysphagia in elderly patients: a retrospective controlled study. Ear Nose Throat J. 2022;103:NP671‐NP678. doi:10.1177/01455613211054631 35255725

[21] Fong TG , Gleason LJ , Wong B , et al. Cognitive and physical demands of activities of daily living in older adults: validation of expert panel ratings. PM R. 2015;7(7):727‐735. 10.1016/j.pmrj.2015.01.018 PMC4508212 25661463

[22] Regan J , Walshe M , Lavan S , et al. Post‐extubation dysphagia and dysphonia amongst adults with COVID‐19 in the Republic of Ireland: a prospective multi‐site observational cohort study. Clin Otolaryngol. 2021;46(6):1290‐1299. 10.1111/coa.13832 PMC8444742 34197688

[23] Martin–Martinez A , Ortega O , Viñas P , et al. COVID‐19 is associated with oropharyngeal dysphagia and malnutrition in hospitalized patients during the spring 2020 wave of the pandemic. Clin Nutr. 2021;41(12):2996‐3006. 10.1016/j.clnu.2021.06.010 PMC8205257 34187698

[24] Brodsky MB , Huang M , Shanholtz C , et al. Recovery from dysphagia symptoms after oral endotracheal intubation in acute respiratory distress syndrome survivors: a 5‐year longitudinal study. Ann Am Thorac Soc. 2017;14(3):376‐383. 10.1513/AnnalsATS.201606-455OC PMC5427721 27983872

[25] Marvin S , Thibeault S , Ehlenbach WJ . Post‐extubation dysphagia: does timing of evaluation matter? Dysphagia. 2019;34(2):210‐219. doi:10.1007/s00455-018-9926-3 30043081

