# Supplementary material for: The characterization of dysphagia in COVID‐19 rehabilitation patients: An inpatient rehabilitation cohort study
Source: PM R. 2026 Apr 9;18(10):1145–53. doi: 10.1002/pmrj.70113 (PMC13686111; doi:10.1002/pmrj.70113)
Supplement: Supplementary file 1 [file PMRJ-18-1145-s001.docx]

| **Finding Domain** | **Key Conclusion** |
| --- | --- |
| **Dysphagia Prevalence** | Highly prevalent at 46.0% (n = 114). |
| **Dysphagia Type** | Oropharyngeal in 89.7%, Oral in 8.6%, Pharyngeal in 1.7% |
| **Dysphagia Resolution** | Dysphagia completely resolved at discharge in 43.5% (n = 62), with 92.1% (n=105) of dysphagia patients on normal or a slightly modified diet of Level 6/0 at discharge. |
| **Dysphagia Development Risk Factors** | FIM Comprehension was significant in adjusted analysis (p = .002) |
| **Dysphagia Resolution Risk Factors** | FIM Comprehension was significant in adjusted analysis (p = .021) |

Table S1: Summary table of the key conclusions from the manuscript.
